# Spontaneous Brain Oscillations Induce Neuronal Excitability Changes That Affect Subjective Perception, Rather Than Decision-Making Strategy

**DOI:** 10.1523/ENEURO.0194-18.2018

**Published:** 2018-06-06

**Authors:** Rosalind S. E. Carney

**Highlighted Research Paper:** Moment-to-Moment Fluctuations in Neuronal Excitability Bias Subjective Perception Rather than Strategic Decision-Making, by Luca Iemi and Niko A. Busch

Spontaneous alpha-band oscillations represent ongoing fluctuations in neuronal excitability, with periods of strong oscillations indicating neuronal inhibition (Watson et al., 2018). Visual detection studies have indicated that heightened neuronal excitability (weak alpha-band oscillations) biases subjects to report seeing a target stimulus even when no stimulus had been presented (Limbach and Corballis, 2016; Iemi et al., 2017). In their *eNeuro* publication, Iemi and Busch (2018) focus on the nature of this bias: is it due to a deliberate preference to report this perception (decision bias), or is it due to a genuine subjective, albeit incorrect, perception of seeing something (perceptual bias)?

They used variations of a two-interval forced choice task, in which subjects were asked to report in which of two intervals a critical stimulus had appeared. The rationale was based on two assumptions. First, bias should facilitate performance on trials in which excitability in the to-be-reported interval happens to exceed excitability in the other interval. Second, while a decision bias (i.e., “I report the interval in which stronger excitability was sensed”) should have that effect irrespective of what the decision is about, a perceptual bias (i.e., “this rather looks like something, as opposed to nothing”) should affect performance only in decisions about stimulus presence versus stimulus absence.

Indeed, Iemi and Busch (2018) found that weak EEG alpha oscillations (strong excitability) before the stimulus-present interval and strong alpha oscillations (weak excitability) before the stimulus-absent interval biased subjects to correctly detect the stimulus in the appropriate interval. No effect of ongoing oscillations was found when the decision was not about stimulus presence versus absence, but instead was about stimulus orientation. This finding implies that fluctuations of neuronal excitability due to ongoing neuronal oscillations in the alpha band amplify sensory signals as well as internal noise, thereby amplifying the subjective impression of seeing something, even when nothing had been presented.

**Figure 1.**
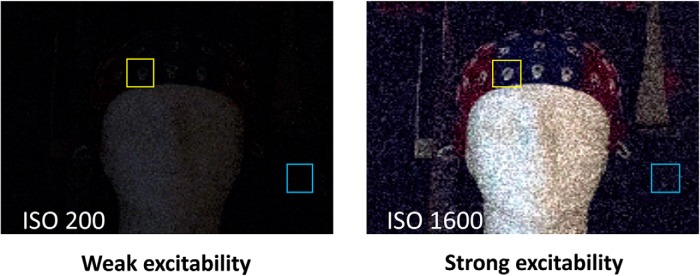
Effect of perceptual bias on perceptual decisions. Left, An image taken at low ISO corresponding to what a subject would see in a brain state of weak excitability. No objects are detected, whether real, but faint (yellow box) or illusory (blue box). Right, An image taken at high ISO corresponding to what a subject would see in a brain state of strong excitability. In this case, even faint objects can be detected (yellow box), but the increased noise level can result in false detection of illusory objects (blue box). (Image courtesy of Niko Busch.).

Conceptually, this perceptual bias can be compared to the effect of the ISO setting (i.e., the sensitivity of a camera to light) (Fig. 1). With low ISO (weak excitability), the camera sensor would not pick up faint objects in the dark; however, low ISO is accompanied by a low noise level. Thus, the photographer might miss an object, but would not mistake the noise for something real. With high ISO (strong excitability), the sensor can pick up even faint objects, but the noise level is also high. Thus, high ISO might “bias” the photographer to see real objects, as well as illusory objects that actually reflect noise.

These results bring up an exciting follow-up question: do these ongoing fluctuations of excitability and perceptual bias, caused by fluctuations in the strength of neuronal oscillations, affect performance in real-world detection tasks such as radiology or airport security screenings?
